# Real-time fluorescence loop-mediated isothermal amplification assay for direct detection of egg drop syndrome virus

**DOI:** 10.1186/s12917-018-1364-9

**Published:** 2018-02-13

**Authors:** Makay Zheney, Zhambul Kaziyev, Gulmira Kassenova, Lingna Zhao, Wei Liu, Lin Liang, Gang Li

**Affiliations:** 1 State Key Laboratory of Animal Nutrition, Institute of Animal Science, Chinese Academy of Agricultural Sciences, Beijing, 100193 People’s Republic of China; 2 Faculty of Veterinary, Kazakh National Agrarian University, Almaty, 050013 Republic of Kazakhstan

**Keywords:** Egg drop syndrome virus, Real-time fluorescence loop-mediated isothermal amplification, Sensitivity, Specificity

## Abstract

**Background:**

Egg drop syndrome (EDS), caused by the adenovirus “egg drop syndrome virus” (EDSV) causes severe economic losses through reduced egg production in breeder and layer flocks. The diagnosis of EDSV has been done by molecular tools since its complete genome sequence was identified. In order to enhance the capabilities of the real-time fluorescence loop-mediated isothermal amplification (RealAmp) assay, we aimed to apply the method for direct detection of the EDSV without viral DNA extraction. In order to detect the presence of the EDSV DNA, three pairs of primers were designed, from the conserved region of fiber gene of the EDSV.

**Results:**

For our assay, test and control samples were directly used in the reaction mixture in 10**-**fold serial dilution. The target DNA was amplified at 65 °C, which yield positive results in a relatively short period of 40–45 min. The method reported in this study is highly sensitive as compared to polymerase chain reaction (PCR) and showed no sign of cross-reactivity or false positive results. The RealAmp accomplished specific identification of EDSV among a variety of poultry disease viruses.

**Conclusions:**

The direct RealAmp can be used to detect the presence of EDSV. As our result showed, the RealAmp method could be suitable for the direct detection of other DNA viruses.

## Background

Egg drop syndrome is a viral disease, caused by the egg drop syndrome virus (EDSV), officially called duck adenovirus 1 (DAdV**-**1), belonging to species Duck adenovirus A, genus *Atadenovirus,* family *Adenoviridae*. EDSV was first reported in 1976, it has also been known as adenovirus 127 and egg-drop-syndrome-76 (EDS**-**76) virus [1]. EDS is characterized by the production of soft-shelled, thin shelled, shell-less, and discolored eggs in otherwise healthy chickens [2]. The natural hosts of the EDSV are ducks and geese, however, the virus can also infect chickens, resulting in major economic losses on egg production [3, 4]. EDSV was involved in severe respiratory disease in 1-day-old goslings where the presence of EDSV DNA was found in different organs of the naturally and experimentally infected goslings [5]. Severe acute respiratory symptoms with coughing, dyspnea, and gasping were reported in 9-day-old Pekin ducklings in 2013 [6]. For diagnosis of EDSV, five serological methods have been used and tested [7]. In the recent years, several PCR studies have been published, for diagnosis of all avian adenoviruses that are of relevance for poultry production [8–10]. Molecular amplification methods were commonly used to diagnose EDSV infection [11].

Loop-mediated isothermal amplification (LAMP) is a method that can amplify DNA under isothermal conditions. It was first developed by the Japanese researchers, the LAMP employs a DNA polymerase and a set of four specially designed primers that recognize a total of six distinct sequences on the target DNA [12]. Later, LAMP was supplemented by using additional primers, termed loop primers which prime strand displacement DNA synthesis. Moreover, LAMP has some advantages in comparison with PCR methods, including improved sensitivity and specificity, as well as time efficiency [13]. Since LAMP was published, a range of LAMP methods have been developed. The RealAmp is one of them, which attempted to improve the method for diagnosis by using a simple and portable device capable of performing both the amplification and detection by fluorescence in one platform [14]. Currently, the LAMP assays are utilized to detect bacterial and viral pathogens including *Mycobacterium tuberculosis*, *Acinetobacter baumannii,* avian influenza virus, Middle East respiratory syndrome coronavirus and hemorrhagic enteritis virus [15–19].

Various LAMP procedures have been successfully employed for DNA amplification using DNA templates extracted from the samples. The purpose of this study was to evaluate the usability of the RealAmp method for a rapid detection of the EDSV in a diverse range of samples without a prior need for nucleic acid extraction. Therefore, we infected both duck embryos and duck fibroblast cell culture with EDSV, then the viral samples were collected and employed to the assay directly by serial dilutions.

## Methods

### Chemicals and reagents

Enzymes including Bst2.0 DNA polymerase (8000 U/ml) and BsrGI-HF (20,000 U/ml) were obtained from New England Bio labs (NEB, USA). Primers for RealAmp and PCR (oligonucleotides) were obtained from Huada (Beijing, China) and suspended in deionized water with appropriate concentrations and stored at − 20 °C. The concentrations of each DNA suspension used in this study were measured by NanoVue Plus spectrophotometer (GE Healthcare, USA).

### Description of the equipment

The fluorescence reader ESE-Quant Tube Scanner used for this study was developed by a commercial manufacturer (ESE Gmbh, Stockach, Germany). It has an eight tube holder heating block with adjustable temperature settings and spectral devices to detect amplified product using fluorescence spectra. This equipment is easy to handle, and completely portable. The results can be seen in a small monitor or on a computer screen connected to the equipment.

### Viruses

Fowl pox virus (FPV isolate FPV4), duck viral enteritis virus (DVEV isolate DPV**-**F37), Marek’s disease virus (MDV isolate CVI 988/Rispens) were obtained from China Institute of Veterinary Drug Control; duck circovirus (DCV isolate DuCV**-**AH1) was obtained from Beijing Experimental Station of Veterinary Biotechnology and Diagnostic Technology, Ministry of Agriculture, China. The viruses were kept in tissue culture supernatant in the laboratory at − 80 °C.

### Inoculation of embryonated duck eggs with the EDSV

For virus propagation 9**-**day**-**old embryonated duck eggs were obtained from Beijing Dayinghongguang Duck Farm, and incubated for 2 days in an egg incubator. EDSV (EDS**-**NE4) used in this experiment was isolated in 1992 [20], and kept in the Animal Disease Control Laboratory of Institute of Animal Science, Chinese Academy of Agricultural Sciences. The virus was diluted with sterile PBS at a ratio of 1/100, followed by inoculation of the allantoic sac of embryonated duck eggs with the viral dilution (200 μL). The eggs were incubated at 37 °C and examined twice each day. After 6 day of incubation the eggs were chilled at 4 °C for 4 h, and then the allantoic fluid was collected from each embryo and stored at − 80 °C. The haemagglutination (HA) titer of EDSV in collected allantoic fluid was in average log_2_12.

### Cell culture and virus inoculation

Duck fibroblast cell cultures were prepared from 11**-**day**-**old duck embryos and cultured in 75 cm^2^ flasks on Dulbecco’s modified Eagle’s medium (DMEM, Gibco, Shanghai, China) supplemented with 10% fetal bovine serum (FBS, Gibco, USA) and 1% gentamycin (Sigma**-**Aldrich). Cells were inoculated with 1 mL of diluted (1:100) EDSV collected from allantoic fluid (HA log_2_12) and incubated at 37 °C under 5% CO_2_ for 1 h. The viral inoculum was removed from the cell layer, and replaced by 10 mL of fresh DMEM supplemented with 2% FBS and 1% gentamycin, followed by incubation for 48 h. PBS was used as a negative control, without virus inoculum in the control flask. The cells were examined daily for any cytopathic effect (CPE). Infected supernatant was harvested after 46 h of incubation and then used for RealAmp analysis. The HA titer of EDSV in harvested cell culture supernatant was log_2_9.

### Viral DNA extraction

Viral DNA was isolated from the allantoic fluid collected from infected duck embryos and the supernatant of duck embryo fibroblasts cultured cells. Total nucleic acids were extracted using the AxyPrep™ Body Fluid viral DNA/RNA Miniprep Kit (Axygen, USA) according to the manufacturer’s instructions and stored at − 20 °C.

### Design of primers for the RealAmp and PCR

**Fig. 1:**
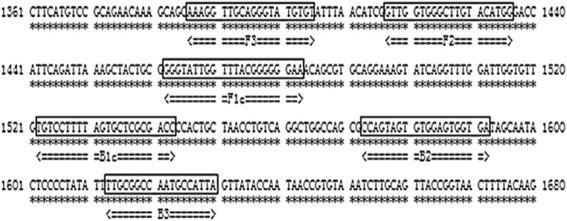
Position of the RealAmp primers in EDSV fiber gene (1935 bp)

Six specific RealAmp primers (F3, B3, FIP, BIP, LF and LB) were designed using PrimerExplorer V5 software (Eiken Chemical Co. Ltd., Tokyo, Japan) based on the fiber gene sequence of the EDSV (GenBank accession No.Y09598.1). The genome positions of RealAmp primers in EDSV genome are shown in Fig. 1. PCR primers were designed using Oligo7 Primer Analysis software (Molecular Biology Insights, Inc. USA). The sequences of the RealAmp and PCR primers are listed in Table 1.

**Table 1:** RealAmp and PCR primers

Method	Primer name	Length (bp)	Sequence (5′-3′)	Location of the primers^a^
RealAmp	F3	20	AAAGGTTGCAGGGTATGTGT	24,070–24,089
	B3	18	TAATGGCATTGGCCGCAA	24,297–24,314
	FIP (F2)	20	GTTGGTGGGCTTGTACATGG	24,101–24,120
	FIP (F1c)	22	TTCCCCCCGTAAACCAATACCC	24,146–24,167
	BIP (B2)	20	TCACCACTCCACACTACTGG	24,257–24,276
	BIP (B1c)	22	TGTCCTTTTAGTGCTCGCGACC	24,206–24,227
	LF	25	CGCAGTAGCTTTAATCTGAATGGTC	24,121–24,145
	LB	20	CCACTGCTAACCTGTCAGGC	24,228–24,247
PCR	Fiber-F	20	ATGAAGCGACTACGGTTGGA	22,685–22,704
	Fiber-R	26	CTACTGTGCTCCAACATATGTAAAGG	24,594–24,619

F3- forward outer primer, B3- backward outer primer, LF- loop forward primer, LB- loop backward primer, FIP- forward inner primer and BIP- backward inner primer. F denotes forward primer and R denotes reverse primer, ^a^locations of primers in EDSV genome

### RealAmp assay

RealAmp reactions were performed using EDSV DNA purified from allantoic fluid and cell culture supernatant. The viral DNA was diluted from 10^− 1^ to 10^− 6^ prior to use. The allantoic fluid and cell culture supernatants were also used directly to the reaction without viral DNA extraction by diluting the samples (10^− 1^ to 10^− 5^). The 25 μL reaction includes 1 μL of template (DNA and virus liquid) in 1X isothermal buffer (Bio Labs, USA; 20 mM Tris–HCl, 50 mM KCl, 10 mM (NH4)_2_SO_4_, 2 mM MgSO_4_, 0.1% Tween 20, pH 8.8) containing 1.2 mM dNTPs, 1 M betaine, 1.6 μM FIP and BIP, 0.2 μM F3 and B3, 0.8 μM LF and LB, and 8 U Bst2.0 DNA polymerase, 9 μL of ddH_2_O and 0.5 μL of EvaGreen (10X) (Invitrogen, Carlsbad, CA). Reactions were carried out at 65 °C, and the total run times were 40–45 min for every RealAmp reaction. The graph of fluorescence units and time was plotted using an ESE**-**Quant Tube Scanner (Qiagen, Germany). The graph shows the fluorescence in millivolts (mV) on the y**-**axis and time in minutes on the x**-**axis. Results can be read in real time using Tube Scanner Studio software.

### Specificity and sensitivity of the RealAmp assay

To validate the specificity of RealAmp for EDSV detection, additional DNA viruses (described in methods section) were tested. To check the expected EDSV targeted RealAmp amplicon, 2 μL of the RealAmp product was digested in 25 μL reaction containing 20 units of BsrGI**-**HF (20,000 U/ml) restriction enzyme, 2.5 μL of Cut-Smart® buffer and 19.5 μL of ddH_2_O. The reaction mixture was incubated at 37 °C for 2 h and separated by electrophoresis in a 1.5% agarose gel.

Sensitivity of the RealAmp assay was tested using 10-fold serial dilutions (10^− 1^ to 10^− 6^) of constructed pMD19T-fiber plasmid DNA (26 ng /μL) and in parallel by conventional PCR method.

### Conventional PCR

Conventional PCR reactions were performed to amplify the fiber gene for construction of pMD19T-fiber plasmid and to compare the established RealAmp sensitivity with conventional PCR. In our study, the size of the target sequence was 245 bp by using the outer primers F3 and B3 and fiber gene 1935 bp by using PCR primers (Table 1). PCR reactions were conducted with a total reaction volume of 25 μL, which contain 12.5 μL of 2X Easy Taq PCR Mix (TransGen; 0.2 mM of each dNTP, 1.5 mM MgCl_2_), 0.4 μM forward primer, 0.4 μM reverse primer, 9.5 μL of ddH_2_O and 1 μL of template DNA. The PCR program consisted of an initial denaturation step at 94 °C for 4 min, followed by 30 cycles of denaturation at 94 °C for 30s, primer annealing at 58 °C for 30s, extension at 72 °C for 2 min (fiber gene primers), 30s for (F3 and B3 primers) and a final extension step at 72 °C for 10 min. PCR products were analyzed by electrophoresis and photographed under UV light.

## Results

### RealAmp of EDSV DNA

**Fig. 2:**
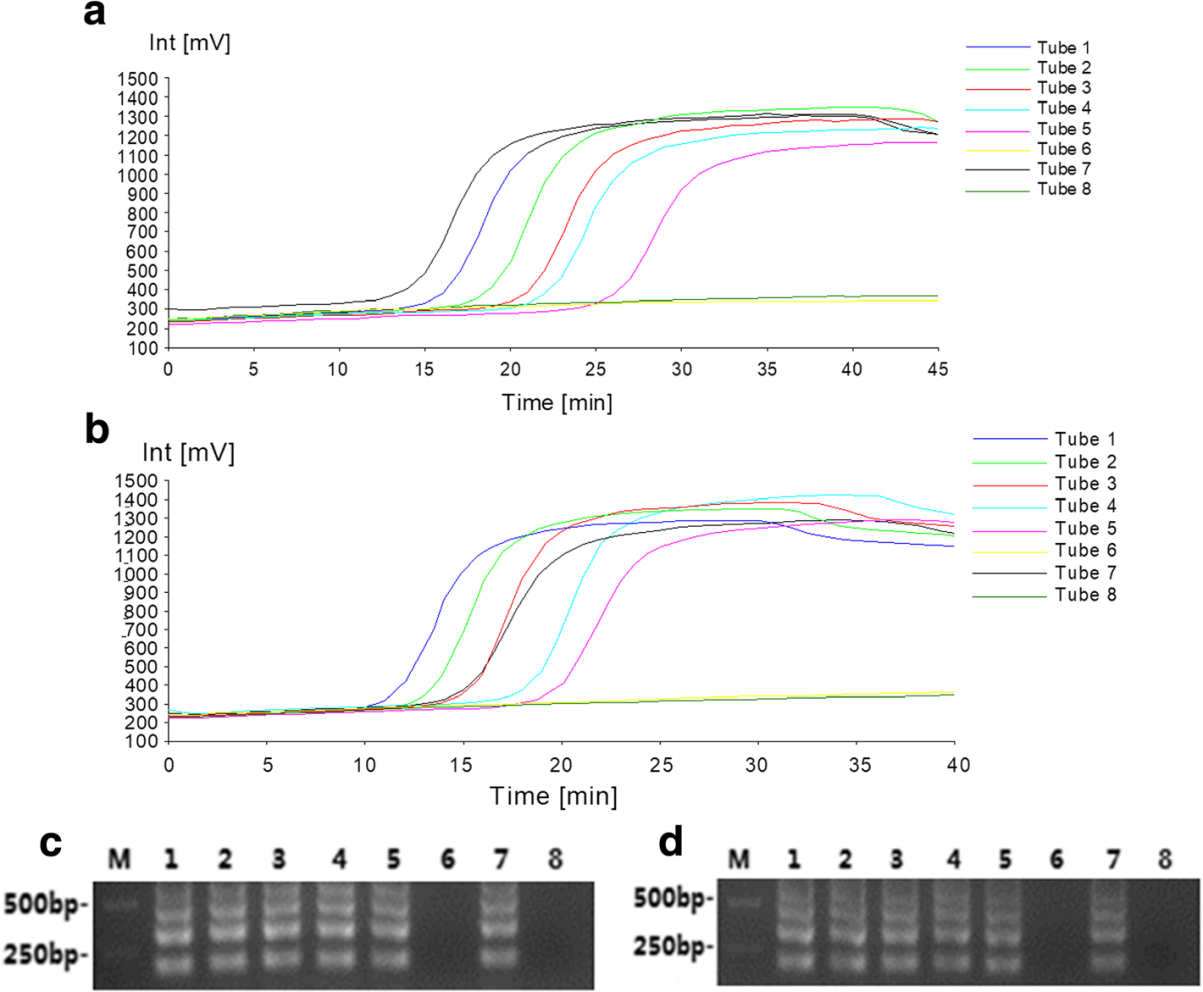
RealAmp using viral DNA isolated from infected allantoic fluid and cell suspension. **a** RealAmp of viral DNA extracted from allantoic fluid; (**b**) RealAmp of viral DNA from infected duck fibroblast cell culture supernatant used with serial dilutions. For both reactions, tube 1–6 DNA sample dilutions (10^− 1^ to 10^− 6^); tube 7 for positive (EDSV DNA) and tube 8 negative (uninfected) controls. **c** and (**d**) 1.5% agarose gel electrophoresis results of (**a**) and (**b**), lane 1–6, sample dilutions (10^− 1^ to 10^− 6^); lane 7 positive control; lane 8 negative control; lane M- Trans2K plus DNA marker

In this assay, the EDSV DNA was successfully amplified from diluted viral DNA samples extracted from infected allantoic fluid and cell culture supernatants within 40 min (Fig. 2a and b). No amplification was obtained from uninfected control samples. The RealAmp reactions were also analysed using agarose gel electrophoresis and as anticipated a ladder**-**like DNA banding pattern was observed (Fig. 2c and d).

### Direct RealAmp assay

**Fig. 3:**
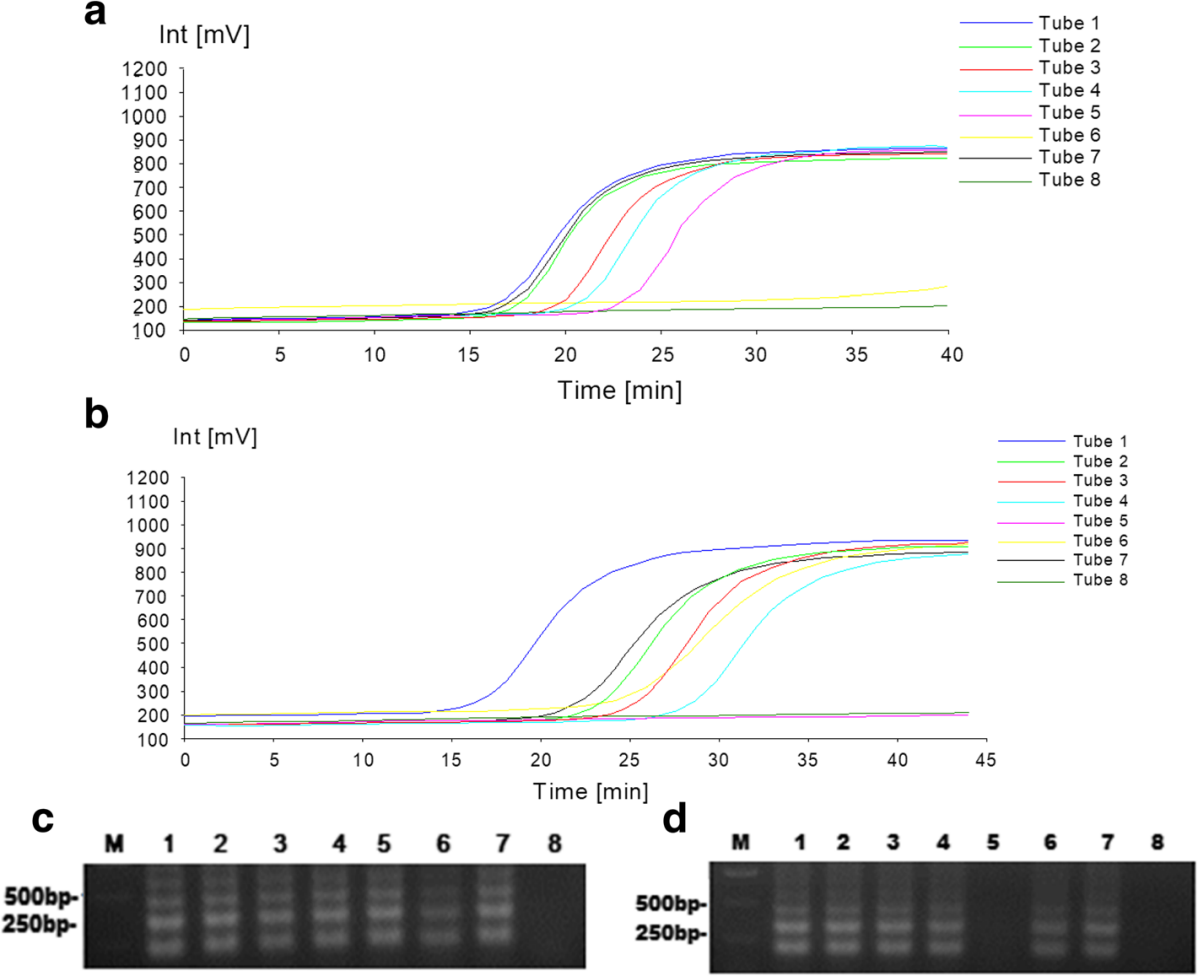
Direct RealAmp assay for allantoic fluid and cell culture supernatant. **a** Infected allantoic fluid; (**b**) cell culture supernatant with serial 10-fold dilutions, tube 1–5 were sample dilutions (10^− 1^ to 10^− 5^); tube 6 undiluted sample; tube 7 positive (EDSV DNA) and tube 8 (uninfected) control. **c** and (**d**) 1.5% Agarose gel electrophoresis results of (**a**) and (**b**), lanes 1–5, sample dilutions (10^− 1^ to 10^−5^); lane 6 undiluted samples; lane 7 positive and lane 8 negative controls. M- Trans2K plus DNA marker

By using infected allantoic fluid and cell culture supernatants, as well as undiluted samples directly in the assay, we successfully amplified EDSV DNA. Analysis of each sample was carried out three times independently. The results obtained were similar to those obtained by using EDSV nucleic acids (Fig. 2). A graph of fluorescence units and time was produced for all sample dilutions. For the allantoic fluid samples, all dilutions from 10^− 1^ to 10^− 5^ showed normal amplification began after 15 min of incubation at 65 °C. However, amplification of the undiluted sample began after 40 min (Fig. 3a). Serial diluted cell culture supernatants (10^− 1^ to 10^− 4^) and undiluted sample provided results after 20–30 min of scanning whereas the 10^− 5^ dilution and uninfected (as a negative control) samples provided no amplification (Fig. 3b). The RealAmp products from both samples were separated in 1.5% agarose gel (Fig. 3c and d).

### Specificity of the RealAmp method

**Fig. 4:**
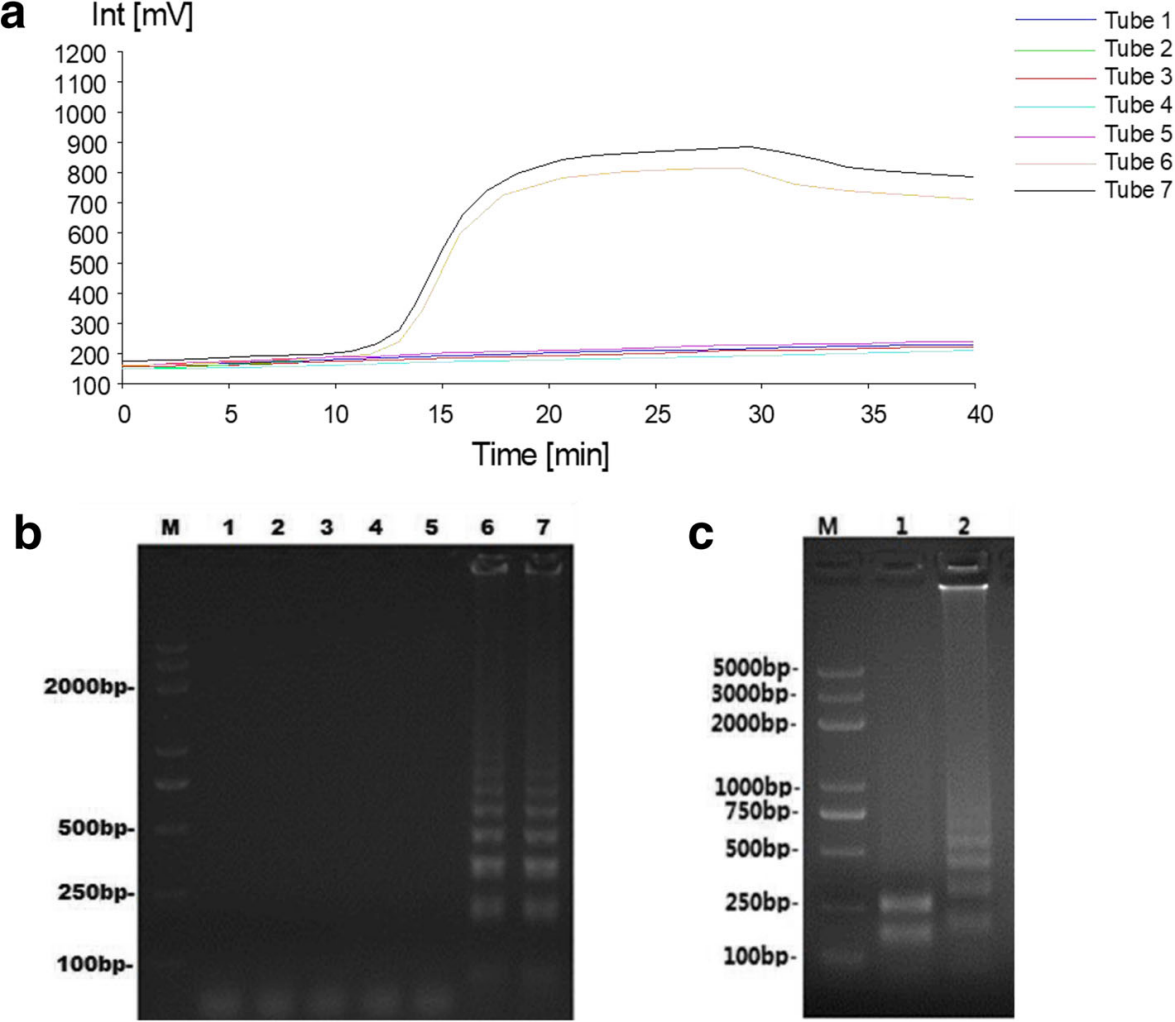
Specificity test of the real-time fluorescence loop mediated isothermal amplification assay for the detection of EDSV. **a** specificity of RealAmp among different virus strains; tube 1 negative control (ddH_2_O); tube 2 FPV; tube 3 DVEV; tube 4 DCV; tube 5 MDV; tube 6 EDSV and tube 7 positive control (EDSV DNA). **b** 1.5% agarose gel electrophoresis of RealAmp products; lane 1 negative control; lane 2 FPV; lane 3 DVEV; lane 4 DCV; lane 5 MDV; lane 6 EDSV and lane 7 positive control. **c** Validation of RealAmp specificity. Lane 1 digested RealAmp product; lane 2 undigested RealAmp product and lane M Trans 2 K plus DNA marker

To evaluate the specificity of the utilized RealAmp assay, we used several poultry disease viruses (described in methods section). As expected, the typical amplification curve was only obtained in the test using EDSV sample as template. The results showed that the RealAmp could amplify and differentiate EDSV gene within other DNA viruses (Fig. 4a and b). The amplified product was digested with restriction enzyme BsrGI**-**HF, which results in the digestion of target region out from a total amplified DNA. The target RealAmp region was cleaved by specific enzyme which is located in the EDSV fiber gene, then separated on a 1.5% agarose gel and shown in Fig. 4c.

### Sensitivity of the RealAmp assay

**Fig. 5:**
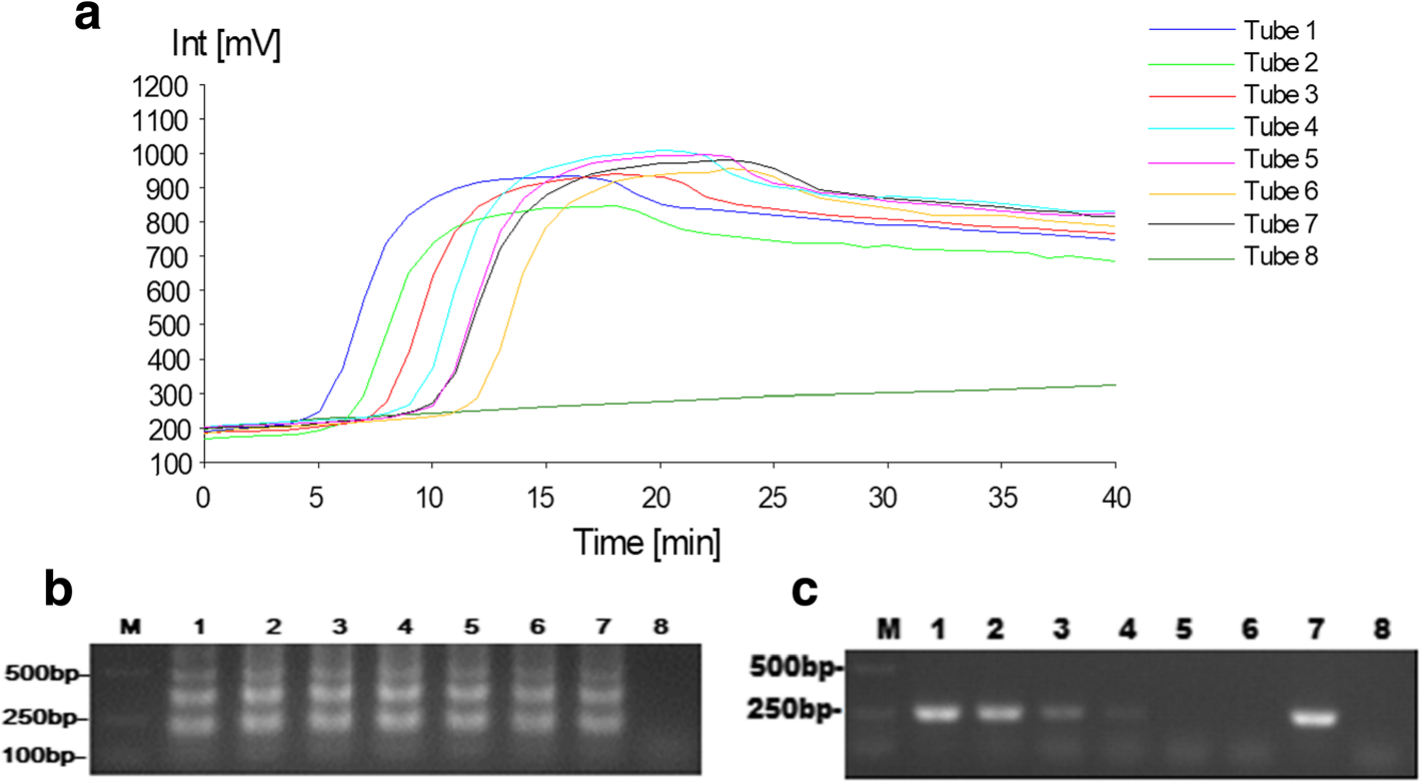
Sensitivity of the RealAmp and PCR. **a** Amplified pMD19T-fiber plasmid DNA by RealAmp (10^− 1^ to 10^− 6^); Reaction 1 2.6 ng; Reaction 2260 pg; Reaction 3 26 pg; Reaction 4 2.6 pg; Reaction 5260 fg; Reaction 6 26 fg; Reaction 7 positive (EDSV DNA) and Reaction 8 negative control (ddH_2_O). **b** 1.5% agarose gel electrophoresis result of RealAmp amplicon. **c** Determination of the detection limit of the PCR with RealAmp outer primers F3 and B3 (Table 1). PCR products were separated in 1.5% agarose gel. Lane 1 2.6 ng; lane 2260 pg; lane 3 26 pg; lane 4 2.6 pg; lane 5260 fg; lane 6 26 fg; lane 7 positive control; lane 8 negative control and lane M Trans2K plus DNA marker

The lower limit of detection of the RealAmp assay determined using plasmid DNA constructed by cloning the PCR amplified fiber gene of EDSV into pMD19T cloning vector (Takara, Japan). The pMD19T-fiber DNA (26 ng) was used to the assay with 10-fold serial dilutions (2.6 ng, 260 pg, 26 pg, 2.6 pg, 260 fg, and 26 fg per microliter). The RealAmp assay demonstrated 100-fold more sensitive than the conventional PCR. The DNA detection limit of the RealAmp was 26 fg and 5.2 × 10^3^ copies/ μL while lower limit of detection of conventional PCR was 2.6 pg and 5.2 × 10^5^ copies/μL. The procedure was monitored using an ESE**-**Quant Tube Scanner (Fig. 5a). Both RealAmp and PCR results were assessed by 1.5% agarose gel electrophoresis (Fig. 5b and c).

## Discussion

Diagnosis of EDS is being performed using molecular technologies in many virus affected countries. The complete genome sequence of egg drop syndrome virus allowed the development of PCR assays for EDSV detection [21]. Since then the hexon based PCR assay was used to detect and differentiate the EDSV from fowl adenoviruses [22]. The conventional PCR methods have been employed in previous studies to diagnose the EDSV infection as a specific and sensitive method as compared to the serological methods [23]. A quantitative real-time PCR (q-PCR) assay based on hexon gene for the rapid detection of EDSV has also been reported [24]. While in 2014 a novel q-PCR assay was used to detect EDSV DNA in samples of interest [25]. Currently, a 151 bp fragment of the EDSV strain 127 penton base gene amplified by PCR with 100% nucleotide identity and confirmed by q-PCR [26]. In the present study, the ESEQuant tube scanner was capable of detecting samples in real-time, and it could analyse melting curves using computer software [14].

To test large number of samples, the real-time PCR methods are too expensive and the q-PCR machines are not always available. Therefore we designed the first EDSV detection by utilizing an assay based on the direct RealAmp. The results of this study clearly indicated the superiority of the RealAmp assay in the detection of EDSV compared to a conventional PCR assay. In order to facilitate improved virus detection, diluted samples of allantoic fluid, and cell culture supernatants were used in the direct RealAmp assay and the results have showed that our method was successfully identified the EDSV from both samples (Fig. 3a and b). The RealAmp cannot give a clear and rapid result, when the sample was highly concentrated (undiluted allantoic fluid sample); the problem lies in the quantity of primer to be much dispersed on different and many DNA pieces. So the self-limiting process could happen (Fig. 3a). In this study, RealAmp has amplified the EDSV fiber gene by using diluted recombinant plasmid DNA as low as 26 fg per microliter in 40 min, while the PCR was around 2.6 pg per microliter in 1 h and 30 min (Fig. 5a and 5c).

## Conclusion

To conclude, the rapid, sensitive and specific RealAmp method can be directly employed to detect EDSV within short time span of 40–45 min in allantoic fluid and cell supernatant by using diluting samples up to 1/10000. Further, this cost effective technique is more sensitive than conventional PCR for detection of EDSV.

## Abbreviations

CPE: Cytopathic effects
DCV: Duck circovirus
DMEM: Dulbecco’s modified Eagle’s medium
DVEV: Duck viral enteritis virus
EDS: Egg drop syndrome
EDSV: Egg drop syndrome virus
FBS: Fetal bovine serum
FPV: Fowl pox virus
HA: Haemagglutination
LAMP: Loop mediated isothermal amplification
MDV: Marek’s disease virus
PBS: Phosphate buffer saline
PCR: Polymerase chain reaction
qRT-PCR: Quantitative real-time polymerase chain reaction
RealAmp: Real-time fluorescence loop mediated isothermal amplification
RT-PCR: Real-time polymerase chain reaction
